# Can Psychosocial Intervention Suppress Testosterone and Triglycerides Among Women With Polycystic Ovary Syndrome? A Feasibility Trial

**DOI:** 10.3389/fpsyg.2021.690539

**Published:** 2021-07-22

**Authors:** Margaret X. C. Yin, L. B. Du, X. N. Zou, Y. L. Fung, Y. Y. Sun, Celia H. Y. Chan, Cecilia L. W. Chan

**Affiliations:** ^1^Department of Social Work and Social Administration, The University of Hong Kong, Hong Kong, China; ^2^Reproductive Medicine Center, The University of Hong Kong-Shenzhen Hospital, Shenzhen, China; ^3^Kids Caring Corner, The University of Hong Kong-Shenzhen Hospital, Shenzhen, China; ^4^Centre on Behavioral Health, The University of Hong Kong, Hong Kong, China

**Keywords:** anxiety, depression, integrative body-mind-spirit (I-BMS), polycystic ovary syndrome (PCOS), testosterone, triglycerides

## Abstract

Women with polycystic ovary syndrome (PCOS) suffer significant psychological distress, which may activate the hypothalamus-pituitary-ovary axis and further affect their physiological state. They often experience elevated levels of testosterone and triglycerides. Considering reports of psychological distress among women with PCOS, this study aimed to develop a psychosocial intervention to improve their emotional and physical health, particularly in Chinese society. This pilot study employed the Integrative Body-Mind-Spirit (I-BMS) intervention model for women with PCOS in China. After a 2 h health information session, 18 participants were randomly assigned to the I-BMS group (9) or the control group (9). The intervention group received 6 weekly, 3 h I-BMS sessions. Pre- and post-blood tests and psychosocial questionnaires were collected from all participants. Retention to treatment was high with 79.6% treatment adherence gained and an overall average of five sessions completed. Compared with the control group, depression and anxiety symptoms reduced significantly for those in the intervention group (*d* = −1.24, *p* < 0.05 and *d* = –1.33, *p* < 0.01), their health-related quality of life improved significantly (*d* = 1.02, *p* < 0.01) both at post-intervention and 3 month follow-up, and their testosterone and triglycerides levels reduced significantly (*d* = −0.97, *p* < 0.001 and *d* = –0.41, *p* < 0.05) after joining the intervention. The I-BMS model is feasible and appears promising in improving psychological health, and reducing testosterone and triglyceride levels, in women with PCOS in China.

**Clinical Trial Registration:** www.chictr.org.cn, identifier ChiCTR1900027606.

## Introduction

Polycystic ovary syndrome (PCOS) is the leading cause of anovulatory infertility, and the most common endocrine disease affecting reproductive-aged women (Balen et al., 2016). The prevalence of PCOS is 10–20% internationally (Naz et al., 2019). Its main clinical manifestations include menstrual irregularity, infertility, obesity, hirsutism, and acne (McCook et al., 2015). The Rotterdam criteria for diagnosing PCOS requires at least two of the three key features to be present: (a) polycystic ovaries on ultrasound; (b) high androgen levels/clinical hyperandrogenism; and (c) menstrual irregularity (Rotterdam Group, 2004). Between 55 and 70% of women suffering from PCOS have elevated testosterone levels compared to healthy women (Huang et al., 2010). Besides sex hormone imbalance, PCOS has been associated with other metabolic complications, such as dyslipidemia, insulin resistance, type II diabetes, and some cardiovascular diseases (Romanowski et al., 2015; Kyrou et al., 2020). PCOS sufferers have been reported as having elevated total cholesterol, triglycerides (TG) and low-density lipoprotein-cholesterol (LDL-C) levels, as well as decreased high density lipoprotein-cholesterol (HDL-C) (Castelo-Branco et al., 2010; Bilal et al., 2018). The etiology of PCOS remains unknown, and there is no current cure (Barthelmess and Naz, 2014).

As PCOS often leads to metabolic and appearance changes, the mental health and mood states of women with PCOS can be affected (Barry, 2019). Barry et al. (2018) described the pathways of testosterone leading to psychological distress, including direct and indirect effects through the unwelcome manifestations of PCOS. The association between dyslipidemia (high total cholesterol, LDL-C or TG, or low HDL-C) and anxiety and depression has also been well presented (Van Reedt Dortland et al., 2013). It has been reported that women with PCOS tend to be more emotionally labile than women who do not suffer from PCOS, and they are more likely to express feelings of anger (Barry et al., 2011a). Research also indicates that PCOS sufferers can experience a range of mental health problems. Stapinska-Syniec et al. (2018) found that 52% of 250 PCOS participants reported depressive symptoms. Significantly more women suffering with PCOS presented with severe anxiety, trait anger, and depression, compared with age-matched healthy women (Erdem et al., 2013). Mood disorders among PCOS sufferers may enhance their disease burden, as those suffering from depression and anxiety may also present with significantly higher dehydroepiandrosterone sulfate (DHEAS) levels (Annagür et al., 2013). Hyperandrogenemia and dyslipidemia in PCOS sufferers have been correlated with anxiety and depression, leading to poor health-related quality of life (HRQoL) (Dokras et al., 2016; Gul et al., 2018).

Chinese women with PCOS have recently been reported as having higher anxiety and depression than healthy women (Yin et al., 2021). This may be because they are distressed by their appearance, or the impact that PCOS may have on their fertility, which hampers their desire to fulfil their filial obligations of continuing the family name in Chinese culture (Yao et al., 2018). To date, there is no known research into psychosocial interventions focusing on holistic mental and physical health for women with PCOS in China.

There is variable evidence of the effectiveness of psychological interventions on physical and mental health of PCOS sufferers. Cooney et al. (2018) conducted a 16-week pilot randomized clinical trial to examine the effect of cognitive-behavioral therapy (CBT) on the mental health of women with PCOS. No significant biometric or metabolic changes were noted at either the week 8 or week 16 assessments. Abdollahi et al. (2018) also used CBT to improve depression among women with PCOS, reporting only slight improvement. Besides CBT, yoga, aerobic training and exercise have also been tested for the care of women with PCOS. The study by Nidhi et al. (2012, 2013a, 2013b) recruited 90 adolescent girls with PCOS, who received a 12-week daily intervention program based on holistic yoga and conventional exercise. Glucose, lipid, insulin values and HRQoL significantly improved after the yoga intervention.

The effectiveness of these interventions may be inconclusive because women with PCOS suffer from complex biological, psychosocial and spiritual pain, and no one intervention was likely to address all these elements. Moreover, although hyperandrogenemia and dyslipidemia have been associated with anxiety, depression and HRQoL, few interventions have targeted all five of these outcome areas. Thus, it is persuasive to employ an integrated method that enhances holistic health and wellbeing, and where intervention effectiveness is tested over a range of parameters.

The Integrative Body-Mind-Spirit (I-BMS) intervention model (Chan et al., 2000) is one such method. It combines Eastern philosophies and therapeutic techniques from Chinese medicine to foster a self-empowerment practice for clients experiencing various social, health and mental health issues. The I-BMS model aims to promote wellbeing of an individual: mind, body and soul. It integrates physical exercises of Tai Chi (also known as taiji), qigong, yoga…., and Eastern philosophic meditations on mindfulness, non-attachment, compassion, forgiveness, gratitude … (Lee et al., 2018). The I-BMS method has been tested on different population groups (e.g., breast cancer sufferers (Liu et al., 2008), women undergoing *in vitro* fertilization (Chan et al., 2012), and in different countries, such as India and Taiwan (Liu et al., 2008; Hsiao et al., 2012; Sreevani et al., 2013). It has consistently produced significant improvements in well-being and holistic health.

The I-BMS model has been adopted as empowerment training for women with PCOS in China, and this pilot study explored its feasibility, and preliminary effects on holistic health. The study aimed to: (1) reduce psychological distress, and improve holistic well-being; and (2) suppress testosterone and dyslipidemia by improving mental health.

## Methods

### Study Design

This was a pilot randomized controlled trial (RCT) examining the effects of the I-BMS intervention on physical and mental health of women with PCOS. A 2 (group: intervention vs. control) × 3 [time: baseline/pre-intervention (T0), post-intervention (T1), 3-month post-intervention (T2)] design was employed. This trial was registered at www.chictr.org.cn (registration number ChiCTR1900027606). The intervention was provided free of charge and participants were compensated for pre/post intervention blood tests.

### Participants

PCOS sufferers were recruited at a Hospital located in Shenzhen, across the border from Hong Kong. Prior to sampling, we distributed an anonymous survey to all women attending the PCOS clinic and then invited selected participants to join the study voluntarily with written informed consent. The diagnosis of PCOS was based on the Rotterdam criteria by the medical team. Women suffering from PCOS who were pre-menopausal, aged 18–35 years old, could understand oral and written Chinese and be physically capable of simple body movements, were recruited by doctors’ referrals. Participants who were eligible and willing to participate provided written informed consent before entering the study. Women who initially agreed but later refused to undergo a full assessment were considered to be dropouts.

Women were excluded if they: (1) had taken oral contraceptives/metformin/insulin-sensitizing treatment, or received pharmacotherapy for dyslipidemia, hypertension, or diabetes/impaired glucose tolerance, or hormonal therapy in the past 4 weeks; (2) were currently suffering from other genetic or endocrine diseases, or neuropsychiatric disorders that require psychotropic substances (such as antipsychotics, antidepressants, or anticonvulsants); (3) were pregnant or who had recently given birth; (4) had participated in similar intervention programs in the past 3 months, or were currently on other exercise training programs or weight loss programs; (5) were smoking on average five or more cigarettes per day, line with the proposed association between nicotine and cortisol levels (Cohen et al., 2004).

### Procedures

This pilot RCT was conducted over 10 months, from September 2019 to June 2020. After doctors’ referrals, potentially relevant individuals joined a pre-group face-to-face interview with the researchers. People’s needs and expectations were collected, and exclusion criteria were applied. Most participants were not familiar with the cardiovascular risks of PCOS. Thus, according to the interviews, a health education information session was designed and the intervention protocol was slightly modified.

All eligible participants were then invited to join a 2 h health education information session. Basic knowledge of PCOS (including manifestations, prevalence and cardiovascular risks) and usual medical treatments were introduced, and brief advice on daily diet and exercise were provided. After the session, all consenting eligible participants were randomly assigned to the intervention or the control group in a 1:1 ratio, by drawing lots. Intervention group participants then joined the I-BMS model program. Control group received no interventions apart from the health education information session.

### Interventions

The intervention consisted of 6 weekly I-BMS sessions which were held on Saturdays at the group counseling center of the hospital. Each session lasted for 3 h. During each session, activities on promoting mind, body, and spiritual wellbeing were shared. The first session was focused on the introduction on mind-body relationship. As PCOS sufferers were concerned about their physical appearance as obtained from the pre-group interviews, the second session tried to help participants to recognize their self-stigma and feelings of shame about their own body. I-BMS methods for self-acceptance and self-compassion were practiced in the third and fourth sessions. The final two sessions facilitated participants to transcend the meaning of the disease, and transform challenges to opportunities. Detailed content of the intervention is provided in Table 1.

**TABLE 1:** Session content of the Integrative Body-Mind-Spirit (I-BMS) group intervention for women with Polycystic Ovary Syndrome (PCOS).

Theme	Purpose	Content
Session I: Listen to your heart: mind-body connection	• Recognize the uncertainty and ambiguity of PCOS • Prepare for body mind adjustment	• Warm-up exercise • Introduction on I-BMS method and holistic health • PCOS emotional problems sharing • Techniques to manage emotions • Body scan • Breathing exercise • Therapeutic massage
Session II: Here and Now: I am beautiful	• Identify objectified experiences • Cultivate healthy values on beauty	• Meditation: feel the nature • Objectified Experience sharing • Introducing objectification • Game: find your own beauty • Self-help acupressure massage
Session III: Self-care and stress management	• Understand the impact of stress • Learn to relieve stress: physical and mental	• Meditation: love and forgiveness • Introducing the nature of stress and its effect on body and mind • Stress and infertility • Experience sharing • Talk and share on ways to reduce stress • Hand massage
Session IV: Self-acceptance and self-compassion	• Learn I-BMS self-acceptance • Accept illness uncertainty	• Meditation: self-acceptance and gratitude • Psycho-education about self-acceptance • Game: self-appreciation and self-compassion • Self-care introduction: developing a healthy lifestyle • Therapeutic massage
Session V: Transformation through loss	• Develop a positive concept of gain and loss • Learn I-BMS skills to transcend losses into deeper meaning in life	• Meditation: regain strength • Introducing psychosomatic mechanism • Sharing on coping process of infertility • Game: Gain and loss transformation • Breathing exercise • Body swinging
Session VI: Empowerment: Building strength and resilience	• Establish a new way of life • Turn losses into resilience • Form a long-term support group to help each other and to promote awareness on PCOS	• Meditation: relaxation and renewal • Re-examining priorities in life • Appreciation of self, others, and life • Future plan sharing • Body exercise skills collection Keep in contact and exercise

### Outcome Measures

All participants provided objective blood tests at T0 and T1, and subjective outcome measures at T0-T2. Besides, feasibility and acceptability were also measured accordingly.

Overnight fasting, morning blood samples were obtained to determine fasting lipid profile (total cholesterol, LDL-C, HDL-C, and TG) and testosterone concentrations. The ratio of TG/HDL-C was also calculated as it may indicate the risk of coronary heart risk (Hadaegh et al., 2009).

Anxiety was measured by the 21-item self-report Beck Anxiety Inventory (BAI). BAI can discriminate anxiety from depression. Each item ranges from 0 to 3; higher scores indicate higher levels of anxiety. The Chinese version of BAI has been validated with excellent reliability and validity (Cheng et al., 2002).

Depression was measured by the 21-item self-report Beck Depression Inventory-II (BDI-II) (Beck et al., 1996). Each item ranges from 0 to 3; high scores correspond to high levels of depression. The Chinese version of BDI-II has been widely adopted with good reliability and validity (Dai and Feng, 2008).

Health-related quality of life (HRQoL) of PCOS sufferers was measured by the Polycystic Ovary Syndrome Questionnaire (PCOSQ). PCOSQ has 26 items, each completed using a 7-point Likert scale. The questionnaire contains five dimensions relating to manifestations of PCOS: hirsutism, obesity, infertility, menstrual irregularity, and emotions. Higher scores indicate better function and less negative influence on daily life. The Chinese version of PCOSQ has been validated demonstrating good reliability and validity (Li et al., 2015).

Feasibility was assessed by testing whether the whole intervention sessions could be completed. If any session could not be completed, the lessons would be summarized, and data analysis would not continue. Besides, screening and enrollment rate, session attendance rate, as well as reasons for absences were recoded.

Acceptability were measured by participants’ post-intervention feedback. After the last session of the intervention, every participant in the intervention group was interviewed for 10–15 min. All participants were asked: (1) did you benefit from the intervention? If yes, how much (from 1 “a few” to 10 “a lot”); (2) which part of the intervention did you remember most? Based on these two questions, participants’ feelings and feedback were collected. Acceptability was also evaluated by any complaints reported to the ethics committee and the dropout rate of the intervention group.

### Data Analyses

Demographics and baseline outcome measures were compared between the intervention and control groups to test for homogeneity (to confirm appropriate randomization). Independent samples *t*-tests or χ^2^-tests were used.

Following intention-to-treat principles, all participants who provided baseline information were included in data analysis, including those who had not adhered to the allocated intervention and those lost to follow-up. Per protocol analysis was also conducted to provide more details. Paired-sample *t*-tests were used to determine within-group effects (T0 vs. T1 and T0 vs. T2). Repeated measures univariate analysis of variance models (ANOVA) (including over-time × group interactions) were constructed to test for between-group differences. The within-group effect size (ES) was calculated by Cohen’s *d* (Cohen, 1988); where values of 0.2, 0.5, and 0.8 were applied to determine small, medium, and large effect sizes, respectively. The between-group ES was calculated by partial eta square (η^2^), which ranges from 0 to 1, with the values of 0.01 being small, 0.08 being medium, and 0.14 being large (Moher et al., 2001).

Pearson’s correlation tests were applied to estimate the association between mental and physical health measures. All data analyses were performed by SPSS 24.0.

### Ethical Considerations

This study was approved by the Medical Research Ethics Committee of the HKU-Shenzhen Hospital (Ethic [2019]167). Personal information was confidential, including names of doctors and nurses in the hospital.

## Results

### Sample

**FIGURE 1:**
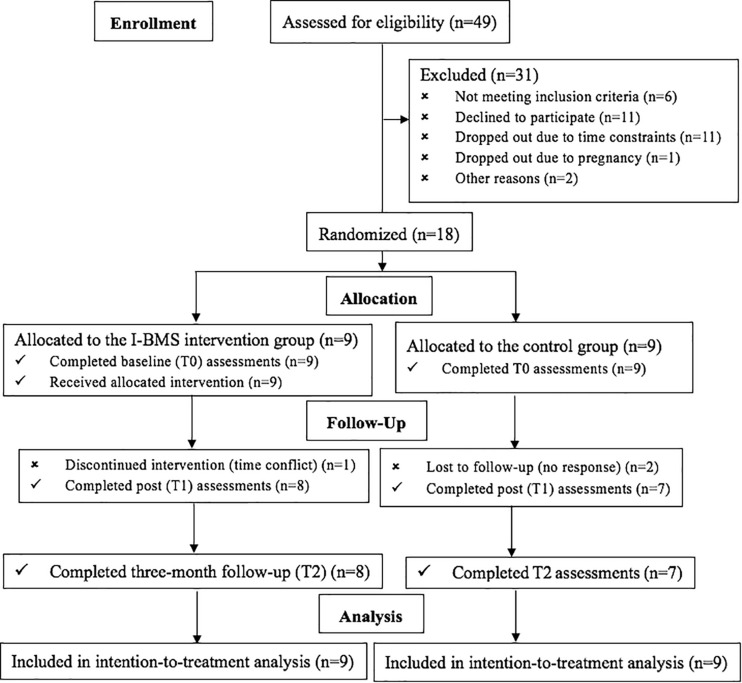
CONSORT diagram of participant flow.

The sampling process is shown in Figure 1. Forty-nine PCOS sufferers were initially screened for eligibility at recruitment, with 18 participants being recruited and randomized: nine into the intervention group and nine into the control group.

### Sample Descriptors and Homogeneity

Socioeconomic characteristics of intervention and control groups are reported in Table 2. Groups did not differ in age, Body Mass Index (BMI) or diagnosis duration (*p* > 0.05). There was no significant difference in marriage, whether have a child or not, education level and monthly income between the two groups. No statistically significant differences were found between intervention and control groups in any mental and physical health factor at T0 (*p* > 0.05).

**TABLE 2:** Socioeconomic characteristics.

Factors	I-BMS group (*n* = 9)			Control group (*n* = 9)			*T*	χ^2^	*p*
	Mean	*SD*	n	Mean	*SD*	n			
Age	29.22	2.33		28.11	3.44		−0.80		0.44
Body mass index	21.25	4.50		21.35	3.38		0.05		0.96
Diagnosed duration	46.56	52.48		43.22	27.29		−0.17		0.87
Marriage								2.10	0.15
Married			7			4			
Unmarried			2			5			
Have a Child								2.25	0.47
No			9			7			
Yes			0			2			
Education level								5.33	0.07
Senior high			1			1			
University			4			8			
Master or above			4			0			
Monthly income								3.34	0.50
3,000–6,000			1			0			
6,000–10,000			1			3			
10,000–20,000			3			4			
20,000–50,000			3			2			
50,000–100,000			1			0			

*I-BMS, Integrative Body-Mind-Spirit; SD, standard deviation.*

### Feasibility

36.7% of the people screened were finally enrolled in the study. The whole six I-BMS intervention sessions were successfully conducted which lasted 175–218 min. Participants in the intervention group attended an average five of six sessions. The overall attendance rate was 79.6%. The reasons for being absent were time conflict with an urgent matter (72.7%) and other reasons (27.3%).

### Acceptability

All participants reported they benefit a lot (seven participants rated 10, one rated 8) from the intervention and they all (100%) thought the intervention was very helpful and should be carried out to more PCOS patients. Six participants thought their internal stress got released. Five participants reflected that they felt more peace and calm after each session. Four participants noticed their mood problems more clearly and learned the I-BMS method to handle them. Two participants described their attitude change toward the program after each session and were grateful to learn about self-acceptance and an optimistic attitude toward the disease and the whole life. No complaints were received to the whole intervention and study. One participant in the intervention group stated her dropout at the end of the second session because of having a busy schedule and refused to take post-intervention measurement. Two participants in the control group lost connection in the post-test. The dropout rate was 11.1% for the intervention group and 22.2% for the control group.

### Treatment Effects

Nine subjects in the intervention group and nine subjects in the control group were included in the intention-to-treat analysis (Tables 3, 4). Eight subjects in the intervention group and seven subjects in the control group were included in the per protocol analysis (Tables 4, 5). Results of intention-to-treat analysis and per protocol analysis were generally similar, but per protocol analysis provided a larger effect size. For brevity, only intention-to-treat analysis results were discussed in the following contents.

**TABLE 3:** Anxiety, depression and health-related quality of life at study time points (Intention-to-treat analysis, *n* = 18).

	Within-group effect								Between-group effect (time × group)						
	T0		T1^a^		ES	T2^a^		ES	T1-T0^b^		T2-T0^b^		*F*	*p*	*ES*
	Mean	*SD*	Mean	*SD*	*d*	Mean	*SD*	*d*	Mean	*SE*	Mean	*SE*			Partial η^2^
**BAI**													3.38	0.06	0.31
I-BMS	8.44	4.56	3.33**	2.96	–1.33	3.78*	3.15	–1.13	−5.11*	1.37	–4.67	1.40			
Control	5.11	4.96	4.44	5.41	–0.13	3.33*	4.87	–0.36	–0.67	1.37	–1.78	1.40			
**BDI**													8.04	0.00	0.52
I-BMS	13.11	8.43	3.89***	3.72	–1.24	4.11**	3.18	–1.40	−9.22**	1.79	–9.00	2.19			
Control	10.44	7.11	9.22	6.12	–0.17	8.00*	6.31	–0.35	–1.22	1.79	–2.44	2.19			
Health Related Quality of Life (PCOSQ)															
**Total**													10.32	0.00	0.58
I-BMS	150.44	19.88	170.33**	19.27	1.02	174.78**	24.29	1.08	19.89***	5.09	24.33*	6.79			
Control	131.78	29.64	118.11**	27.57	–0.47	134.67	33.68	–0.09	–13.67	5.09	2.89	6.79			
**Hirsutism**													2.64	0.10	0.26
I-BMS	32.00	2.78	33.33	2.06	0.51	33.89	2.21	0.75	1.33*	1.55	1.89*	1.59			
Control	27.22	7.93	23.56	10.21	–0.38	26.00	9.50	0.14	–3.67	1.55	–1.22	1.59			
**Acne**													3.06	0.08	0.29
I-BMS	24.78	3.46	26.22	2.91	0.45	26.44	2.79	0.52	1.44*	1.19	1.67*	1.22			
Control	18.00	8.99	15.67	7.50	–0.26	17.89	8.09	–0.01	–2.33	1.19	–0.11	1.22			
**Obese**													2.38	0.13	0.24
I-BMS	24.22	10.85	28.33	8.44	0.40	28.00*	9.14	0.34	4.11*	1.67	3.78	1.31			
Control	19.00	8.00	18.00	7.53	–0.13	20.89	10.03	0.18	–1.00	1.66	1.89	1.31			
**Infertile**													1.39	0.28	0.16
I-BMS	9.89	4.86	11.56	3.25	0.39	13.67	4.80	0.78	1.67	1.18	3.78	1.44			
Control	11.11	5.16	10.33	4.64	–0.15	11.67	4.74	0.11	–0.78	1.18	0.56	1.44			
**Menstrual**													1.07	0.37	0.13
I-BMS	25.44	4.75	28.44	6.54	0.43	29.11	4.26	0.75	3.00	2.15	3.67	1.76			
Control	21.89	6.09	20.67	5.79	–0.20	22.56	5.22	0.11	–1.22	2.15	0.67	1.76			
**Emotion**													5.88	0.01	0.44
I-BMS	34.11	11.33	42.44*	5.00	0.82	43.67*	8.52	0.94	8.33**	2.65	9.56	3.01			
Control	34.56	10.68	29.89	8.36	–0.46	35.67	11.68	0.10	–4.67	2.65	1.11	3.01			

BAI, Beck Anxiety Inventory; BDI, Beck Depression Inventory-II; PCOSQ, Polycystic Ovary Syndrome Questionnaire; SD, standard deviation; ES, effect size; SE, standard error. ^a^Within-group difference using paired t-test. ^b^Between-group difference using independent t-test. *p < 0.05; **p < 0.01; ***p < 0.001.

**TABLE 4:** Pre- and post-intervention tests of physical biomarkers.

	Intention-to-treat analysis (*n* = 18)										Per protocol analysis (*n* = 15)									
	Within-group effect					Between-group effect (Time × group)					Within-group effect					Between-group effect (Time × group)				
				
	Pre-intervention (T0)		Post-intervention (T1)^a^		ES	T1-T0^b^		F	*p*	ES	Pre-intervention (T0)		Post-intervention (T1)^a^		ES	T1-T0^b^		*F*	*p*	*ES*
														
	Mean	*SD*	Mean	*SD*	*d*	Mean	SE			η^2^	Mean	*SD*	Mean	*SD*	*d*	Mean	*SE*			η^2^
**Body mass index**								4.37	0.05	0.21								4.00	0.07	0.24
I-BMS	21.25	4.50	20.92	4.39	−0.08	−0.34*	0.12				21.56	4.71	21.18	4.62	−0.08	−0.38*	0.13			
Control	21.35	3.38	21.35	3.37	0.00	0.00	0.12				20.61	1.65	20.61	1.62	0.00	0.00	0.14			
**Testosterone (ng/mL)**								23.08	0.000	0.59								29.00	0.000	0.69
I-BMS	0.72	0.19	0.54***	0.18	−0.97	−0.18***	0.03				0.71	0.20	0.51***	0.17	−1.08	−0.20***	0.03			
Control	0.77	0.24	0.79	0.26	0.08	0.02	0.03				0.69	0.19	0.71	0.23	0.10	0.02	0.03			
**Total cholesterol (mmol/L)**								1.36	0.27	0.08								1.77	0.21	0.12
I-BMS	4.75	0.66	4.67	0.57	−0.12	−0.08	0.17				4.73	0.71	4.64	0.60	−0.14	−0.09	0.19			
Control	4.47	1.09	4.12	0.72	−0.26	−0.35	0.17				4.72	1.06	4.27	0.67	−0.51	−0.45	0.20			
**TG (mmol/L)**								7.91	0.01	0.33								8.18	0.01	0.39
I-BMS	1.01	0.43	0.83*	0.35	−0.41	−0.17*	0.07				1.06	0.42	0.87*	0.36	−0.49	−0.20*	0.08			
Control	0.88	0.31	0.98	0.36	0.28	0.10	0.07				0.86	0.33	0.99	0.40	0.36	0.13	0.08			
**HDL**-**C (mmol/L)**								0.04	0.85	0.00								0.03	0.87	0.00
I-BMS	1.51	0.34	1.54	0.29	0.09	0.03	0.07				1.50	0.36	1.53	0.31	0.09	0.03	0.08			
Control	1.52	0.49	1.53	0.48	0.02	0.01	0.07				1.58	0.50	1.59	0.48	0.02	0.01	0.08			
**LDL**-**C (mmol/L)**								0.55	0.47	0.03								0.92	0.36	0.07
I-BMS	3.19	0.67	3.02	0.57	−0.27	−0.17	0.13				3.19	0.72	3.00	0.61	−0.29	−0.20	0.15			
Control	2.82	1.24	2.51	0.90	−0.17	−0.31	0.13				3.05	1.23	2.64	0.84	−0.39	−0.40	0.16			
**TG/HDL**-**C**								5.90	0.03	0.27								5.80	0.03	0.31
I-BMS	0.73	0.44	0.58*	0.33	−0.32	−0.15*	0.06				0.78	0.44	0.61*	0.34	−0.43	−0.05	0.16			
Control	0.65	0.34	0.70	0.33	0.16	0.06	0.06				0.58	0.25	0.65	0.26	0.27	0.20	0.19			

TG, triglycerides; HDL-C, high density lipoprotein-cholesterol; LDL-C, low-density lipoprotein-cholesterol; SD, standard deviation; ES, effect size; SE, standard error. ^a^Within-group difference using paired t-test. ^b^Between-group difference using independent t-test. *p < 0.05; ***p < 0.001.

**TABLE 5:** Anxiety, depression and health-related quality of life at study time points (Per protocol analysis, *n* = 15).

	Within-group effect								Between-group effect (time × group)						
	T0		T1^a^		ES	T2^a^		ES	T1-T0^b^		T2-T0^b^		*F*	*p*	*ES*
	Mean	*SD*	Mean	*SD*	*d*	Mean	*SD*	*d*	Mean	*SE*	Mean	*SE*			Partial η^2^
**BAI**													3.25	0.07	0.35
I-BMS	8.25	4.83	2.50*	1.69	–1.59	3.00*	2.27	–1.39	−5.75**	1.52	−5.25*	1.55			
Control	6.29	5.02	5.43	5.80	–0.16	4.00*	5.39	–0.44	–0.86	1.63	–2.29	1.66			
**BDI**													9.01	0.00	0.60
I-BMS	13.63	8.86	3.25**	3.41	–1.56	3.50*	2.78	–1.55	−10.38***	1.86	−10.13**	2.37			
Control	11.57	7.53	10.00	6.53	–0.22	8.43*	6.93	–0.43	–1.57	1.99	–3.14	2.53			
**Health Related Quality of Life (PCOSQ)**															
**Total**													13.42	0.00	0.69
I-BMS	151.50	20.98	173.88**	17.19	1.17	178.87**	22.40	1.08	22.38**	5.20	27.38**	7.56			
Control	135.43	31.12	117.86**	29.66	–0.58	139.14	35.69	0.11	−17.57*	5.56	3.71	8.08			
**Hirsutism**													3.23	0.08	0.35
I-BMS	32.25	2.87	33.75	1.75	0.63	34.38	1.77	0.89	1.50	1.72	2.13	1.85			
Control	25.29	7.99	20.57	9.59	–0.54	23.71	9.62	–0.18	–4.71	1.84	–1.57	1.97			
**Acne**													3.80	0.05	0.39
I-BMS	25.38	3.16	27.00	1.85	0.63	27.25	1.49	0.76	1.63	1.34	1.88	1.43			
Control	18.00	8.62	15.00	6.27	–0.40	17.86	7.34	–0.02	–3.00	1.44	–0.14	1.53			
**Obese**													2.38	0.13	0.28
I-BMS	25.38	10.99	30.00	7.27	0.50	29.62*	8.26	0.44	4.63	1.90	4.25*	1.46			
Control	22.00	6.11	20.71	6.02	–0.21	24.43	8.22	0.34	–1.29	2.03	2.43	1.56			
**Infertile**													1.23	0.33	0.17
I-BMS	9.88	5.19	11.75	3.41	0.43	14.12	4.91	0.84	1.88	1.37	4.25	1.64			
Control	12.00	5.48	11.00	5.00	–0.19	12.71	4.79	0.14	–1.00	1.46	0.71	1.76			
**Menstrual**													0.96	0.41	0.14
I-BMS	25.00	4.87	28.38	6.99	0.56	29.13	4.55	0.88	3.38	2.51	4.13	2.03			
Control	22.43	4.58	20.86	4.18	–0.36	23.29	2.56	0.23	–1.57	2.68	0.86	2.17			
**Emotion**													6.59	0.01	0.52
I-BMS	33.63	12.01	43.00*	5.04	1.02	44.37*	8.82	1.02	9.38*	2.91	10.75*	3.40			
Control	35.71	12.01	29.71	9.60	–0.55	37.14	13.03	0.11	–6.00	3.11	1.43	3.63			

BAI, Beck Anxiety Inventory; BDI, Beck Depression Inventory-II; PCOSQ, Polycystic Ovary Syndrome Questionnaire; SD, standard deviation; ES, effect size; SE, standard error. ^a^Within-group difference using paired t-test. ^b^Between-group difference using independent t-test. *p < 0.05; **p < 0.01; ***p < 0.001.

Considering within-group effect, the mean value of participants’ depression symptoms reduced significantly (*p* < 0.001, *d* = −1.24) at T1 from moderate depression (13.11 ± 8.43) to no symptoms (3.89 ± 3.72). This effect lasted at T2 (*p* < 0.01, *d* = −1.40). There was a significant decrease in anxiety (*p* < 0.01, *d* = − 1.33), testosterone (*p* < 0.001, *d* = −0.97), TG (*p* < 0.05, *d* = −0.41), and TG/HDL-C (*p* < 0.05, *d* = −0.32) and a significant increase in emotion-related quality of life (*p* < 0.05, *d* = 0.82) and total HRQoL (*p* < 0.01, *d* = 1.02) in the intervention group at T1. The effect sizes at T1 were all medium to high according to Cohen’s *d* estimates. The intervention effects on anxiety (*p* < 0.05, *d* = −1.13), emotion-related quality of life (*p* < 0.05, *d* = 0.94) and total HRQoL (*p* < 0.01, *d* = 1.08) also lasted at T2. Besides, the effect on obese-related quality of life became significant at T2 (*p* < 0.05, *d* = 0.34).

The between-group effect analysis showed a significant time × group interaction in depression (*F* = 8.04, *p* < 0.01, η^2^ = 0.52), and testosterone (*F* = 23.08, *p* < 0.001, η^2^ = 0.59), TG (*F* = 7.91, *p* < 0.05, η^2^ = 0.33) and TG/HDL-C (*F* = 5.90, *p* < 0.05, η^2^ = 0.27), meaning that over time depression, testosterone, TG and TG/HDL-C significantly decreased in the intervention group than the control group. The interaction was also significant in emotion-related quality of life (*F* = 5.88, *p* < 0.05, η^2^ = 0.44) and total HRQoL (*F* = 10.32, *p* < 0.01, η^2^ = 0.58) meaning that emotion-related QoL and total HRQoL increased in the intervention group but decreased in the control group over time. The effect sizes η^2^ ranged from moderate to high.

### Mind-Body Associations

**FIGURE 2:**
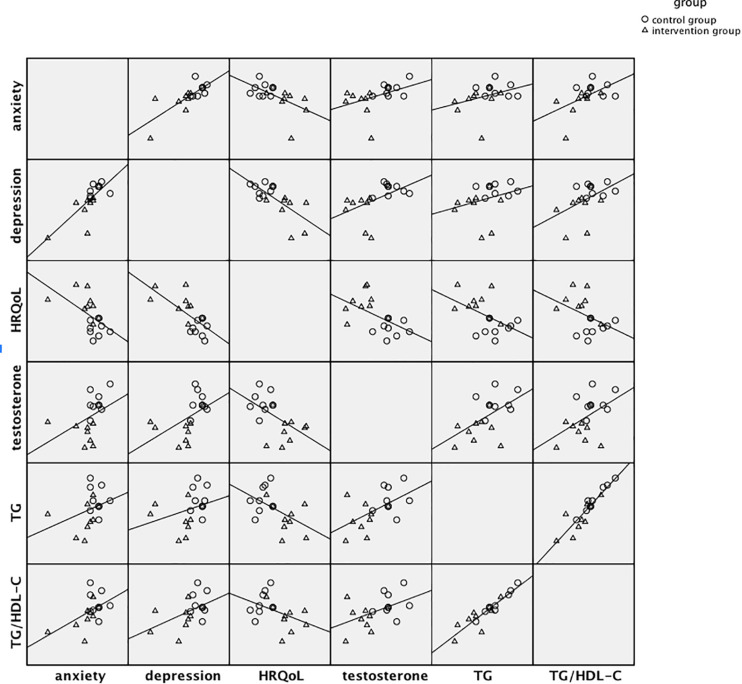
Scatterplot matrix of associations between standardized residuals of pre-post changes in major mental and physical factors.

The pre-post (T1-T0) changes in anxiety, depression, HRQoL, testosterone, TG and TG/HDL-C were calculated to examine the associations among them. Significant associations were found between changes of anxiety and depression (*r* = 0.77, *p* < 0.001), anxiety and HRQoL (*r* = −0.56, *p* < 0.05), anxiety and TG/HDL-C (*r* = 0.52, *p* < 0.05), and changes of depression and HRQoL (*r* = −0.69, *p* < 0.01), depression and testosterone (*r* = 0.51, *p* < 0.05), depression and TG/HDL-C (*r* = 0.49, *p* < 0.05). There were also significant associations between changes of HRQoL and testosterone (*r* = −0.54, *p* < 0.05), HRQoL and TG (*r* = −0.51, *p* < 0.05). Figure 2 showed the scatterplot matrix of associations between standardized residuals of changes in each outcome, which presented the mind-body associations and the significant intervention effect.

## Discussion

This pilot study suggested that for this small group of randomly assigned Chinese PCOS sufferers, the I-BMS intervention model improved mental and physical health, and holistic well-being compared to the control group. To the best of our knowledge, this is the first study to employ an integrative non-pharmacological intervention for Chinese PCOS patients, and the first study to examine the effect of a mind-body psychosocial intervention on testosterone and dyslipidemia of PCOS patients. The results were in line with previous findings of I-BMS interventions for other Chinese populations, supporting the interconnectivity among body and mind, and verifying the efficacy of the non-pharmaceutical I-BMS model in reducing mental and physical distresses (Chan et al., 2017). Moreover, previous psychological interventions for PCOS patients produced only limited benefits, yet the I-BMS model brought definite physical and mental improvements. Besides good feasibility, the participants in the intervention group gave positive feedback about the intervention, which helped verify its acceptability. Thus there is sufficient support from this pilot study to proceed to a larger RCT to test I-BMS applications for Chinese PCOS patients.

The mean depression and anxiety scores reduced significantly after the intervention ceased and at 3 month follow-up, showing a relatively high effect size. Mean testosterone, triglycerides and ratio of triglycerides/high-density lipoprotein cholesterol improved significantly more for the intervention group than the control group, indicating that the intervention group were functioning better after completing the 6 week intervention program. Mean scores for emotion-related QoL and total HRQoL also improved significantly in the intervention group at T1 and T2 compared to the control group, with moderate or high effect sizes. These results supported our hypothesis that the I-BMS model is likely to effectively reduce Chinese PCOS sufferers’ anxiety, depression, testosterone and triglycerides, and improve their HRQoL.

The study results were in line with previous research on the relationship between hyperandrogenemia, dyslipidemia and depression and anxiety (Nese et al., 2011; Baskind and Balen, 2016). In our study, prior to being diagnosed with PCOS, most participants reported experiencing stressful events which resulted in worsening bodily and ovarian function. This supports the negative influence of psychosocial stress and anxiety on ovarian function and hypothalamic–pituitary axis (Baskind and Balen, 2016), and on neuroendocrine and metabolic system of PCOS patients (Nese et al., 2011). The intervention fostered a happy, positive and resilient attitude toward life. As depression and anxiety symptoms improved, patients’ testosterone and triglyceride levels decreased. This supports Barry et al.’s (2018) hypothesis regarding the pathway from psychology to biology. Despite the fact that total cholesterol levels did not change significantly, the study validated the interrelationship between physiological (i.e., hyperandrogenism, triglyceride, TG/HDL-C), and psychological (i.e., depression, anxiety) factors among women with PCOS (Farrell and Antoni, 2010; Cooney and Dokras, 2017).

This pilot study has certain limitations. Firstly, the sample size was small. Although the doctors referred 49 patients during the 3 months of recruitment, only 18 participants were successfully randomized. Considering the dropout reasons and feedback from successful participants, this finding reflects potential future difficulties in the development of clinical group counseling in Chinese contexts. Secondly, all participants were not particularly over-weight nor with obvious appearance changes. Thus, modifications may be needed for future studies to adopt I-BMS to be relevant for more PCOS patients with diverse characteristics.

PCOS has been consistently associated with mental health conditions such as anxiety, depression and lowered HRQoL (Amini et al., 2012; Chaudhari et al., 2018). Metabolic changes incur bodily distress as well as psychiatric symptoms (Barry et al., 2011b); self-image dissatisfaction negatively influences mental health status, resulting in emotional frustrations (Scaruffi et al., 2019). Besides CBT, there are few psychological interventions that have been developed specifically for women with PCOS. Moreover, as the disease has no cure, self-efficacy is an important aspect of management. The I-BMS model offers a holistic empowerment intervention (Lee et al., 2018). After the program, intervention group participants were empowered to practice the self-care exercises of emotion self-management, self-acceptance and self-care, healthy lifestyle, meaning-making, acupressure, abdominal breathing, and other I-BMS techniques by themselves. Early indications from this pilot study are that I-BMS is a suitable support for women who are adjusting to living with PCOS for life. The convincing effect sizes provide the basis for calculating a robust sample size for a larger RCT to further understand how I-BMS works for women suffering from PCOS.

## Conclusion

In conclusion, this pilot study suggests that the I-BMS intervention is likely to be more effective for Chinese women with PCOS, compared to other intervention methods, in improving physical and mental health symptoms. This indicates that larger-scale research is feasible to test this intervention in Chinese PCOS sufferers.

## Data Availability Statement

The original contributions presented in the study are included in the article/supplementary material, further inquiries can be directed to the corresponding author/s.

## Ethics Statement

The studies involving human participants were reviewed and approved by the Medical Research Ethics Committee of the HKU-Shenzhen Hospital. The patients/participants provided their written informed consent to participate in this study.

## Author Contributions

MXCY conducted the intervention and drafted the original manuscript. LBD and XNZ helped in the recruitment and measurement design, and gave advice to revise the manuscript. XNZ, YLF, and YYS gave advice on the intervention protocol and feasibility test. CHYC gave advice on the intervention protocol and revised the manuscript. CLWC gave some advice on the manuscript. All authors contributed to the article and approved the submitted version.

## Conflict of Interest

The authors declare that the research was conducted in the absence of any commercial or financial relationships that could be construed as a potential conflict of interest.
